# Associations between workplace bullying and later benefit recipiency among workers with common mental disorders

**DOI:** 10.1007/s00420-021-01764-1

**Published:** 2021-10-11

**Authors:** Camilla Løvvik, Simon Øverland, Morten Birkeland Nielsen, Henrik Børsting Jacobsen, Silje Endresen Reme

**Affiliations:** 1 Department of Psychosocial Science, University of Bergen, Bergen, Norway; 2 Division of Mental and Physical Health, Norwegian Institute of Public Health, Bergen, Norway; 3 Haukeland University Hospital, Bergen, Norway; 4 Department of Pain Management and Research, Oslo University Hospital, Oslo, Norway; 5 Department of Psychology, Faculty of Social Sciences, University of Oslo, Oslo, Norway; 6 National Institute of Occupational Health, Oslo, Norway

**Keywords:** Common mental disorders, Work participation, Sickness absence, Disability, Workplace bullying, Social support

## Abstract

**Objective:**

In this study, we examined exposure to workplace bullying as a predictor of registry-based benefit recipiency among workers struggling with work participation due to common mental disorders. Further, we examined if the experience of receiving social support moderated the association between workplace bullying and benefit recipiency.

**Design:**

Secondary analyses of a randomized controlled trial.

**Patients:**

People struggling with work participation due to common mental disorders (CMD).

**Methods:**

Study participants (*n* = 1193) were from a randomized controlled trial (The At Work and Coping trial (AWaC), trial registration http://www.clinicaltrials.gov NCT01146730), and self-reported CMD as a main obstacle for work participation. Participants were at risk of sickness absence, currently on sickness absence or on long-term benefits. Benefit recipiency indicated sickness absence and/or long-term benefits (i.e., disability pension) at 6-month follow-up.

**Results:**

Of the 1193 participants, 36% reported exposure to workplace bullying. Workplace bullying was significantly associated with benefit recipiency at 6-month follow-up (OR 1.41, CI 1.11–1.79). Social support did not moderate the association between bullying and benefit recipiency.

**Conclusions:**

The finding that workplace bullying increases the risk of later benefit recipiency suggest that bullying is a significant obstacle for work participation.

**Supplementary Information:**

The online version contains supplementary material available at 10.1007/s00420-021-01764-1.

## Introduction

Exposure to bullying at the workplace is detrimental for health (Verkuil et al. 2015) and is also an important risk factor for expulsion from working life (Niedhammer et al. 2013). Targets of bullying consistently report reduced work ability due to both mental and somatic health complaints (Nielsen and Einarsen 2012; Verkuil et al. 2015; Leach et al. 2020; Lever et al. 2019; Romero Starke et al. 2020; Niedhammer et al. 2021; Nielsen et al. 2020; Xu et al. 2019), higher levels of sick leave (Niedhammer et al. 2013; Nielsen et al. 2016; Eriksen et al. 2013; Lesuffleur et al. 2014), and increased risk of disability retirement (Nielsen et al. 2014; Glambek et al. 2015; Berthelsen et al. 2011) compared to colleagues not exposed to bullying. At the same time, common mental disorders (CMD) is a major reason for work disability across the OECD countries (OECD 2012). Barriers for return to work in those on sick leave due to CMD operate on different levels, including on the individual, workplace, healthcare, and societal level (Reme 2020). While workplace bullying is an individual experience that is largely determined by the perceptions of those exposed, it exists within the workplace and is a particularly strong barrier for return to work, with a dose–response relationship to adverse outcomes (Ortega et al. 2011).

Workplace bullying involves exposure to negative acts from one or several others occurring repeatedly and in situations where the target of these actions finds it difficult to defend him- or herself (Einarsen et al. 2011). Distinctive to workplace bullying is also the tendency for these actions to escalate, and to involve a perceived imbalance in power between the perpetrator(s) and the victim(s) (Einarsen et al. 2011). Workplace bullying is therefore not about single episodes of conflict or harassment, but rather a form of systematic harassment where the exposed employee is unable to handle or end the exposure (Einarsen 2000; Nielsen and Einarsen 2018).

It has been suggested that bullying impacts on health and well-being of those exposed by breaking down assumptions of themselves as valuable and worthy individuals, of other people as benevolent, and of the world as meaningful (Mikkelsen 2001; Mikkelsen and Einarsen 2002). Such abrupt changes in core conceptual beliefs is threatening and can inflict psychological crisis resulting in mental disorders (Janoff-Bulman 1992). The effect of such exposures may be mitigated by protective factors. Significant moderation effects have been found for hardiness (Reknes et al. 2018), job autonomy and occupational self-efficacy (Livne and Goussinsky 2018) on mental health outcomes, whilst social support has demonstrated effects on sick leave as well (Nielsen et al. 2020). Social support therefore appears particularly relevant to investigate as a protective factor in a context of people with CMD aiming to return to work.

What’s more, CMD and workplace bullying are associated in a bi-directional manner, implying that a person with a CMD is more likely to experience workplace bullying (Verkuil et al. 2015). To the best of our knowledge, no studies have looked specifically at workplace bullying in relation to sickness absence within this population. We believe that this is an important knowledge gap that needs to be addressed. Following this, the aims of this study were to: (1) evaluate if workplace bullying reported by people with CMD predicted benefit recipiency at 6-month follow-up, and (2) if social support moderated the impact of workplace bullying on benefit recipiency at 6-month follow-up. Through these aims, this study will be the first to determine the predictive role of workplace bullying, and the moderating role of social support, in workers exposed to workplace bullying who concomitantly struggle with work participation due to CMD.

## Methods

### Design

This is a secondary analysis of a randomized controlled trial of 1193 participants struggling with work participation due to CMD. The aim of the trial was to evaluate the effect of work-focused cognitive behavioural therapy (CBT) and an adaptation of Individual Placement and Support (IPS) on work participation (The At Work and Coping trial (AWaC) (Reme et al. 2015). The trial was commissioned by the Norwegian health and welfare authority, which specifically asked for an experimental evaluation of an intervention scheme (AWaC) in a real-world setting. The trial was therefore designed as a pragmatic, multicentre randomized controlled trial.

### Procedure

All the centres in the country offering the AWaC program were defined as a research project during the trial period. Accordingly, the AWaC program could only be accessed through trial participation in the trial period. Participants were recruited through referrals from the national insurance office (NAV), primary care physicians and self-referrals. After trial recruitment ended, we continued the data collection to compare the study population with those who sought the same services outside the trial. We found similar characteristics across both populations (Overland et al. 2016), indicating a low risk of selection bias. The main inclusion criterion for the trial was current struggles with work participation, with CMDs as the main perceived obstacle. Persons indicating other reasons as the main obstacle for work participation, like social, financial or any work-related reasons (including psychosocial workplace factors), would not be considered eligible for participation. Clinical psychologists conducted the assessment of CMDs based on self-reports of symptoms consistent with anxiety and/or depression. This included sub-threshold symptoms of anxiety and depression disorders. In addition to CMD as main obstacle for work participation, other important inclusion criteria were; age 18–60 years, no severe psychiatric illness or suicide risk, no ongoing substance abuse, no ongoing individual psychotherapy outside of the trial and an explicit commitment to either maintain work participation or initiate the return-to-work process. Study participants were recruited regardless of sickness absence status and could as such be at risk of sickness absence, currently on sickness absence or on long-term benefits when entering the trial. At risk of sickness absence was defined by the patients through self-report and self-referrals, and it included those who struggled with work participation but were still either fully at work or combining work and sick leave.

Prior to inclusion, a brief screening procedure (30 min) was administered to assess eligibility according to predefined inclusion criteria and to provide participants with detailed information about the trial. Informed consent was signed by those willing and eligible, and the right to withdraw from the study at any point was emphasized. During the inclusion period (2010 to 2012), there were 1416 potential participants referred and considered for inclusion. Of these, 197 did not fulfil the inclusion criteria, 17 did not consent to participate, and 9 with- drew their consent and required data deletion (see Supplementary file 1 flow chart for details). All participants completed comprehensive baseline questionnaires at the time of inclusion. Questionnaire packages contained, among others, measures of mental and somatic health complaints, various demographic variables, and other social and work-related characteristics. Results from the trial showed no effects of the intervention on work participation at 6-month follow-up. The complete results from the main trial have been presented elsewhere (Reme et al. 2015).

Study design and procedures were approved by the Regional Ethics Committee (reference number: 2010/1130) and done according to the Helsinki declaration.

### Measures and variables

In this study, we applied self-report data from baseline questionnaires (clinical and demographic participant characteristics, exposure to workplace bullying, perceived social support) and objective data from national registries on sickness absence and benefits at baseline and 6-month follow-up (work status at baseline and benefit recipiency at baseline and follow-up).

#### Workplace bullying

Exposure to workplace bullying was measured at baseline by asking participants: “Have you ever been bullied at work (including both current and former work places)”? In this study, the term exposure to workplace bullying, thus resembles what is also sometimes referred to as being a self-labelled victim of workplace bullying (Vie et al. 2012). The measure of workplace bullying used in this study is somewhat similar to other measures of workplace bullying without time constraints (Saunders et al. 2007), and to the measure used by Asfaw et al. (2014) to examine the relationship between workplace bullying and sickness absence in the U.S. Responses to the workplace bullying item were given on a five-point scale with the alternatives “never”, “on rare occasions”, “from time to time”, “once a week” or “several times a week”. We chose to dichotomize workplace bullying the following way; “never been bullied at work” (coded as “0”) and “have been bullied at work” (“on rare occasions” or “from time to time”, “once a week” or “several times a week”, coded as “1”). This dichotomization is in line with previous studies on workplace bullying (Berthelsen et al. 2011; Nielsen et al. 2009).

#### Social support

The experience of social support was assessed at baseline with four items constructed within the research group, asking about whether the respondents had been talking to someone within or outside the family about “joys and sorrows” and “health issues” during the last 14 days. i.e., “During the last 14 days, have you talked to someone within the family about joys and sorrows?” and “During the last 14 days, have you talked to others, except the family, about health issues?” Response categories were “1—Yes” and “2—No”. The responses were recoded and summarized into an overall indicator of social support with higher scores indicating higher levels of support.

#### Benefit recipiency

Objective data from the National Insurance Register in Norway were used to construct variables for ascertaining benefit recipiency at baseline and 6-month follow-up. These national registries contain the complete and objective information on participants’ recipiency of health-related benefits from the national welfare service including disability pension, work assessment allowance, unemployment benefits and sickness benefits. All participants gave individual consent to have data on benefit recipiency retrieved from these registries. For the purpose of this study, those in our study population who received such benefits were coded as “1”, while those who did not were coded as “0”. Access to objective data from registries ensured complete follow-up (100%) on our outcome variable. Due to the heterogeneity of sickness absence statuses in this study population, the predicted outcome (benefit recipiency) would indicate different transitions dependent upon participants’ sickness absence status at baseline. For those at risk of sickness absence at baseline, the outcome indicated a transition from work to sickness absence or other long-term benefits, whereas for those on sickness absence or long-term benefits at baseline the outcome indicated that the participant was still receiving benefits at follow-up and thus had failed to attain or resume work participation.

### Statistical analysis

Statistical analyses were conducted with SPSS 24.0. As the outcome variable (benefit recipiency) was dichotomous, analyses of main and interaction effects were conducted using logistic regression analyses. Binary logistic regression analysis was used to examine if exposure to workplace bullying predicted benefit recipiency at 6-month follow-up. The two-way interactive effect between the predictor variables was tested by adding an interaction term between bullying and social support (e.g., bullying × social support) to the logistic regression. All variables included in logistic regression models were checked for multicollinearity and interaction effects prior to inclusion in the main analysis. Previous studies have shown that both workplace bullying (Glambek et al. 2018; Cuyper et al. 2009) and disability recipiency is related to age (Knudsen et al. 2012). Age and gender were therefore included as control variables in all adjusted analyses.

## Results

Overall, there were more female (67%) than male participants, the mean age was 40.4 years, a majority had education at university or postgraduate levels (60.5%). At baseline, 31.4% of the participants were working (of these, 48% were combining work and sick leave), 39% were fully on sick leave, 21.7% were in long-term benefits (> 12 months sick leave) and 7.9% were unemployed (included in the subgroup “long-term benefits”). Altogether 48% of the sample were benefits recipients at the 6-month follow-up. An overview of clinical and demographic characteristics is presented in Table 1. Frequency distributions showed that of 1193 participants in the overall sample, 36% (*n* = 430) reported exposure to workplace bullying, while 64% (*n* = 752) of the study population had never been bullied. Of those that reported previous workplace bullying, 12% (*n* = 146) had been bullied “on rare occasions”, 15% (*n* = 182) “from time to time”, 1% (*n* = 13) “once a week” and 7.5% (*n* = 89) “several times a week”.

**Table 1:** Overview of clinical and demographic characteristics in study sample

	Total (*n* = 1193)	Exposed to workplace bullying (*n* = 430)
*Continuous variables*	Mean (SD)	Mean (SD)
Age	40.4 (9.7)	40.6 (9.5)
Hospital Anxiety and Depression Scale (HADS)		
Total score	18.7 (6.9)	19.9 (6.6)
Illness duration (years)	8.6 (9.7)	10.1 (10.6)
Self-reported health status (1–5)^1^	2.7 (0.8)	2.8 (0.8)
*Categorical variables*	*n* (%)	*n* (%)
Gender		
Female	800 (67.1)	295 (66.1)
Education		
University/postgraduate college	722 (60.7)	251 (58.6)
Civil status (married/cohabitant)	648 (54.4)	218 (50.7)
Blue collar workers	391 (32.8)	143 (33.3)
Baseline work status		
At risk of sickness absence	334 (28.0)	107 (24.9)
On sickness absence	529 (44.3)	182 (42.3)
On long-term benefits	330 (27.7)	141 (32.8)
Mental health status, HADS, (cut off = > 8)		
Anxiety	926 (78.2)	353 (82.9)
Depression	633 (53.5)	270 (63.4)

^1^Lower score indicates better self-reported health status

Across the three subgroups of benefit recipients at baseline, exposure to workplace bullying was reported in 32.1% (*n* = 250) of those at risk of sickness absence, 38.3% (*n* = 285) of those on sickness absence, and 38.6% (*n* = 215) of those on long-term benefits. The slightly higher prevalence of bullying in the “long-term benefits group” was not significantly different from the other groups (*p* > 0.05). As a sensitivity analysis, we ran stratified analyses in the sub-group closest to work and the sub-group furthest away from work. We found identical ORs in both sub-groups (at-risk of sickness absence: OR 1.41, CI = 0.87–2.28, long-term benefits: OR: 1.41, CI = 0.78–2.54).

The distribution of social support as reported by the participants were as follows: During the last 14 days, 77.8% (*n* = 924) reported that they had talked to someone within the family about joys and sorrows, 67% (*n* = 793) reported that they had talked to someone within the family about health issues, 73.6% (*n* = 872) reported that they had talked to someone outside the family about joys and sorrows, and 62.8% (*n* = 744) reported that they had talked to someone outside the family about health issues. The count variable reflecting the sum score of these four items indicated that 6.9% (*n* = 81) responded negative to all four items, 9.9% (*n* = 117) responded positive to one of the items, 23.1% (*n* = 272) responded positive to two of the items, 15.4% (*n* = 181) responded positive to three of the items, and 44.6% (*n* = 525) responded positive to all four items.

Workplace bullying (OR 1.41 (CI 1.11, 1.79)) was a statistically significant predictor of benefit recipiency at 6-month follow-up. The association between exposure to bullying and benefit recipiency remained statistically significant also after adjusting for age and gender.

Table 2 presents the findings from the analysis of the main interactive effect of bullying and social support on benefit recipiency, adjusted for age and gender. The analyses of main effects showed that exposure to bullying (OR 1.34 (CI 1.05, 1.70)) and age (OR 0.98 (CI 0.97, 0.99)), but not social support (OR 0.94 (CI 0.86, 1.03)) and gender (OR 1.21 (CI 0.94, 1.55)), were significantly associated with likelihood of benefit recipiency at the 6-month follow-up. The test of the interactive effect between bullying and support was not significant (OR 1.05 (CI 0.88, 1.25)), indicating that perceived social support did not moderate the impact of bullying on later benefit recipiency.

**Table 2:** Main and interactive effects (two-way) of bullying and overall score on social support on benefit recipiency 6 months later (*n* = 1193)

Step	Variable	B	SE	OR	95% CI
1					
	Age	− 0.02	0.01	0.98**	0.97–0.99
	Gender	0.19	0.13	1.21	0.94–1.55
	Bullying	0.29	0.12	1.34*	1.05–1.70
	Social support	− 0.06	0.05	0.94	0.86–1.03
2					
	Age	− 0.02	0.01	0.98**	0.97–.99
	Gender	0.19	0.13	1.20	0.94–1.55
	Bullying	− 0.03	0.62	0.97	0.29–3.27
	Social support	− 0.08	0.06	0.92	0.82–1.03
	Bullying*social support	0.05	0.09	1.05	0.88–1.25

*p < .05; **p < .01

## Discussion

The findings from this study show that more than one third of respondents from a population struggling with work participation due to CMD had been exposed to workplace bullying (36%). The findings further showed that workplace bullying is a significant risk factor for subsequent benefit recipiency, as previous targets of bullying had a 41 percent increased risk of receiving benefits after 6 months when compared to non-targets. In contrast to expectations, perceived social support did not moderate the association between bullying and subsequent benefit recipiency.

A substantial proportion of this sample reported exposure to workplace bullying. Whereas the prevalence rate of bullying in the general Norwegian working population is about 5 percent (Nielsen et al. 2009), 36 percent of the respondents in the current sample reported to have been bullied at work. Although this finding shows that exposure to bullying is prevalent among benefit recipients, it should be noted that some of the disparities in frequency may be due to methodological issues. While the abovementioned study by Nielsen and colleagues presented respondents with a formal definition of workplace bullying before asking about exposure, bullying was here measured with a single item question, without providing participants with a definition of the construct. Previous studies suggest that assessing bullying without providing a definition tends to return higher frequencies (Nielsen et al. 2009), most likely due to increasing false positive reports in the absence of a formal definition of bullying, (Nielsen et al. 2011). We did not apply any time constraint to our measure of workplace bullying, and may therefore have picked up exposures not related to current mental health or work-related problems, potentially leading to an underestimation of the true relationship. Nevertheless, the finding that workplace bullying is a significant risk factor for later disability recipiency is in line with previous research on associations between bullying and participation in working life. Targets of workplace bullying have previously been found to express intentions to leave and to change employers more often than non-bullied workers (Berthelsen et al. 2011), and targets are also at higher risk of long-term sickness absence (Nielsen et al. 2016; Kivimäki et al. 2000; Grynderup et al. 2017) and disability retirement (Nielsen et al. 2017; Glambek et al. 2015). For instance, Niedhammer et al. (2013) linked exposure to workplace bullying with both occurrence, and duration of sickness absence across different worker populations, albeit in a cross-sectional study. A recent prospective observational study also found exposure to workplace bullying to be a risk factor for long-term sick leave (Ortega et al. 2011). A possible explanation for the finding is that the lack of control victims of bullying experience, their limited possibility for escape, as well as the power imbalance between perpetrator and victim, make a prolonged time away from work among the few viable coping strategies to resort to.

Despite our theoretical reasons for expecting social support to function as a buffer in the association between workplace bullying and subsequent benefit recipiency, the results provided no indications for such a protective effect of social support. Hence, this finding goes against a previous study that found that social support moderated the indirect association between workplace bullying and sickness absence through mental distress (Nielsen et al. 2019). One explanation for why we found no effect of social support in this study may be that we did not differentiate between specific sources of social support. While we utilized a measure of general social support without relating this to the workplace, Nielsen and colleagues examined support from three different sources: leaders, colleagues, and persons outside the workplace. The findings showed that only colleague and leadership support were beneficial with regard to reducing the impact of bullying. This suggests that work-related social support is particularly important, and that our findings may have been different if we had examined social support from work-related sources. However, with regard to the findings by Nielsen and co-workers, it should be noted that the protective effects of colleague and leadership support on the effects of bullying actually could reflect an implementation of measures and interventions against bullying. If so, it may not be the social support per se that is protective, but instead that the social support reported is in fact a proxy for actual actions taken to stop the bullying.

### Study strengths and limitations

The outcome, benefit recipiency, was based on data from objective and complete national registries on sickness absence and disability benefits enabling complete follow up on all participants, thus representing a considerable study strength. The pragmatic design of the trial contributes to a high external validity by mimicking usual conditions of care. To examine this question as it pertains to generalizability of findings outside the context of a trial, we repeated the baseline questionnaire in a sample that were recruited into the AWaC intervention after the trial recruitment was ended. The participant characteristics between the trial participants and those who sought the same services outside the trial were similar which should strengthen the generalizability and applicability of results outside the trial context (Overland et al. 2016). This supports that the findings are relevant for the target group of the trial: people who struggle with work participation due to common mental disorders. Despite these strengths, there are also some limitations to this study. As the indicators of bullying, social support, and mental distress were self-report measures, the findings may be influenced by problems that are common to self-report methodology, such as response set tendencies. With regard to the measure of workplace bullying included in this study, we did not include a formal definition of workplace bullying. Hence, it could be that each participant responded with his or her own subjective, and perhaps unique, definition of workplace bullying in mind. Providing respondents with a formal definition of workplace bullying has previously been found to produce more conservative prevalence estimates (Nielsen et al. 2010). However, in Scandinavian countries, the prevalence of workplace bullying across studies with or without a formal definition have also been found not to differ (Saunders et al. 2007). Nevertheless, we suggest that future studies on workplace bullying as predictors of work participation, such as benefit recipiency, would benefit from including validated measures and formal definitions of workplace bullying to be able to directly compare findings across studies.

## Conclusions

There is still much to learn about obstacles for work participation. Those already struggling to maintain their work participation, for instance due to CMD, may have been exposed to adverse events in working life, such as workplace bullying. The prevalence rates of bullying were very high in the present study population, and significantly associated with increased risk of being on benefit recipiency (sickness absence and/or disability) 6 months later. As discussed, one explanation for the negative impact of bullying on workability is that exposure to bullying shatters the target’s basic assumptions about themselves as worthy individuals, other persons as benevolent, and the world as meaningful. While preliminary evidence supports such an explanation (Mikkelsen and Einarsen 2002; Rodriguez-Munoz et al. 2010; Adoric and Kvartuc 2007), more research is needed to fully establish the role of basic assumptions. With regard to return-to-work interventions, it is also important to establish measures that can contribute to re-establish the target’s workability. The findings of this study did not support a protective effect of social support in the relationship between bullying and work participation. Still, we did not measure social support at the workplace specifically, which could have given other results. Nevertheless, the high frequency of workplace bullying along with the higher risk of later work disability amongst those exposed to bullying, adds support to that bullying prevention may reduce sick leave and disability rates.

## Supplementary Information

Below is the link to the electronic supplementary material.

Supplementary file1 (DOCX 41 KB)

## Data Availability

The registry data are not available due to law regulations. The survey data are available upon reasonable request.
