# Supplementary material for: Associations between workplace bullying and later benefit recipiency among workers with common mental disorders
Source: Int Arch Occup Environ Health. 2021 Oct 11;95(4):791–8. doi: 10.1007/s00420-021-01764-1 (PMC9038823; doi:10.1007/s00420-021-01764-1)
Supplement: Supplementary file 1 — Supplementary file1 (DOCX 41 KB) [file 420_2021_1764_MOESM1_ESM.docx]

Allocated to intervention (n=630)

Allocated to control group (n=563)

Lost to follow-up for primary outcome (n=0)

Lost to follow-up for primary outcome (n= 0)

## Follow-Up

## Allocation

Randomized (n=1202)

( (n= 573)

Assessed for eligibility (n= 1416)

197 not eligible according to inclusion criteria (multiple reasons in 47 cases) - 13 not within age range - 76 Other than mental health problems obstructing work participation - 64 not motivated to initiate RTW process within 4-6 weeks - 45 severe mental illness - 44 ongoing psychological therapy elsewhere - 4 pregnancy - 1 substance abuse - 3 not fluent in Norwegian

## Enrollment
